# Predator presence versus predator cues: oviposition trade-offs in *Aedes albopictus*

**DOI:** 10.1093/jme/tjag134

**Published:** 2026-08-07

**Authors:** Noga Luna Wasserberg, Gideon Wasserberg, Alon Silberbush

**Affiliations:** Department of Physics and Astronomy, North Carolina State University, Raleigh, NC, USA; Department of Biology, University of North Carolina at Greensboro, Greensboro, NC, USA; Department of Biology and the Environment, Oranim Academic College, Kiryat Tivon, Israel

**Keywords:** oviposition, predator cues, conditioned water, microbial volatiles, predation risk assessment

## Abstract

Predator presence can influence mosquito oviposition decisions through direct and indirect cues, yet most studies have examined indirect chemical cues alone. We evaluated the relative effects of live *Gambusia affinis* fish (direct cue) versus fish-conditioned water (indirect cue) on oviposition by container breeding mosquitoes (mostly *Aedes albopictus*) in a field experiment conducted at the University of North Carolina at Greensboro. Paired oviposition cups (treatment and control) were deployed along a transect during three replicate 1-wk sessions for each treatment. Egg numbers were analyzed using paired *t*-tests, and oviposition responses were quantified using the Oviposition Activity Index (OAI). Live fish significantly deterred oviposition. More eggs were laid in control cups (43.2 ± 5.7) compared to cups containing fish (25.9 ± 8.2; OAI = −0.45). When analyses were restricted to cups in which fish remained present throughout the experimental period, deterrence was even stronger (OAI = −0.63). In contrast, fish-conditioned water significantly increased oviposition relative to aged-water controls (47.5 ± 9.1 vs. 30.2 ± 6.0; OAI = 0.20). These contrasting responses suggest that gravid *A. albopictus* females discriminate between immediate predation risk and residual environmental cues associated with prior predator presence. We hypothesize that attraction to fish-conditioned water may be mediated by microbially derived volatiles stimulated by fish excretions, whereas avoidance of live fish likely involves visual or mechanical cues. Our findings suggest that oviposition site selection reflects a trade-off between cues indicating a high-quality habitat and offspring survival risk, highlighting the multimodal nature of predator detection in container-breeding mosquitoes.

## Introduction

The presence of predators in aquatic habitats is a major factor influencing the populations and communities of prey species in these systems (Resetarits Jr. and Wilbur 1989, Blaustein et al. 1995, Wellborn et al. 1996). Predators can affect prey both numerically through direct consumption and indirectly by inducing behavioral or life-history changes (Preisser et al. 2005, Weissburg 2012, Weiss et al. 2012). In small-volume aquatic habitats, such as ponds or containers, even a few predators can strongly influence the survival of adult prey or their offspring. This is particularly critical for organisms that lack parental care and have limited larval dispersal, where oviposition site selection is a key fitness-enhancing decision (Resetarits Jr. and Wilbur 1989, Blaustein et al. 1995, Wellborn et al. 1996, Weissburg 2012). For these organisms, the “Preference-Performance Hypothesis” predicts that natural selection favors oviposition behavior that maximizes offspring performance (Thompson 1988). Accordingly, gravid females are expected to avoid laying eggs in sites containing larval predators. The ability to detect and avoid predator-containing sites is well documented in amphibians and aquatic insects (Resetarits Jr. and Wilbur 1989, Blaustein 1999, Kershenbaum et al. 2012). Mosquito larvae have a wide variety of potential predators, including predatory insects (eg *Toxorhynchites* larvae, dragonfly nymphs, hydrophilic beetles and backswimmers), crustaceans (eg copepods), and amphibians (eg bufonid and salamander tadpoles), as broadly summarized in several reviews of predator effects on mosquito ecology (Blaustein 1999, Juliano 2009, Becker et al. 2010). In many of these systems, studies have shown that gravid female mosquitoes can detect larval predators and modify oviposition site selection, accordingly, often avoiding habitats that pose elevated predation risk to their offspring (Blaustein 1999, Silberbush et al. 2010, Vonesh and Blaustein 2010, Day 2016).

Larvivorous fish are the most efficient predators of mosquito larvae, in comparison to other larvivorous species, and have long been used as biological control agents in a wide range of aquatic habitats (Becker et al. 2010, Griffin and Knight 2012). The consumptive effects of larvivorous fish, particularly *Gambusia affinis*, have been extensively documented, with numerous studies demonstrating substantial reductions in mosquito larval abundance and, in some cases, adult mosquito populations (Walton 2007, Pyke 2008). In contrast, the non-consumptive (behavioral) effects of larvivorous fish have received considerably less attention. Most studies examining these effects have found that female mosquitoes preferentially avoid ovipositing in water containing larvivorous fish. This predator avoidance behavior has been documented across multiple mosquito genera, including *Culiseta* (Silberbush 2022), *Aedes* (Ritchie and Laidlaw-Bell 1994, Pamplona et al. 2009, Rathnayaka et al. 2021), and *Anopheles* (Petranka and Fakhoury 1991), although the magnitude of the response varies among species.

In *Culex* mosquitoes, eg, oviposition avoidance from *G. affinis* (Baird and Girard)-conditioned water has been repeatedly documented and is stronger than responses to other fish such as Green Sunfish (Eveland et al. 2016, Silberbush and Resetarits Jr. 2017) and iridescent tooth carp (Cohen and Silberbush 2021), suggesting species-specific predator recognition. Nonetheless, mosquito responses to fish are not universal and may vary according to their typical oviposition habitat. For instance, van Dam and Walton (2008) conducted laboratory choice assays to test how fish exudates affected oviposition by *Aedes aegypti*, *Culex quinquefasciatus*, and *Culex tarsalis*. The 2 *Culex* species typically oviposit in open-water habitats such as ponds and puddles. Both species clearly avoided ovipositing in fish-conditioned water, whereas the container-breeding mosquito *A. aegypti* showed no such avoidance. This observation led to the hypothesis that the absence of oviposition avoidance in *A. aegypti* reflects an adaptive response to settings where encounters with larvivorous fish are not likely (eg small natural or artificial containers). Conversely, other studies have reported negative responses of *A. aegypti* and *Aedes albopictus* to exudates from several fish species (Rathnayaka et al. 2021). It is important to note that most studies on this topic evaluated indirect indicators of fish presence (fish exudates). In one study that evaluated direct fish-presence effects (Alcalay et al. 2019), both *Culex pipiens* and *Culiseta longiareolata* laid significantly more egg rafts in fish-free pools than in adjacent pools containing caged fish, although the avoidance response was considerably stronger in *C. pipiens* than in *C. longiareolata*. In a second study (Segev et al. 2017), *Culex* mosquitoes also avoided ovipositing in habitats containing caged fish. The strength of this response varied among fish species, suggesting species-specific predator avoidance. The only study evaluating the effect of live fish on container breeding mosquitoes was done by Pamplona et al. (2009), who showed, under lab settings, that *Betta splendens* fish but not *Poecilia reticulata* significantly reduced the oviposition response of *A. aegypti* gravid females.

In this study, we conducted a field study to evaluate the effects of the presence of *G. affinis* fish, representing a direct cue, with those of fish-conditioned water, representing an indirect cue, on the oviposition response of container-breeding mosquitoes. Considering the natural history of these mosquitoes that typically do not encounter fish in their small natural or artificial breeding containers, we did not anticipate a significant effect of either fish presence or fish-conditioned water on oviposition site response (Vonesh and Blaustein 2010).

## Materials and Methods

The study was conducted between 2 September and 14 October 2020, at the Peabody-Park, University of North Carolina at Greensboro. The “direct cue” experiment (live fish) consisted of three 1-wk replicate sessions (2 September to 23 September), followed by 3 replicate sessions assessing the “indirect cue” effect of fish-conditioned water (30 September to 14 October). For each experiment, we used a single 120 m long transect comprising 6 pairs of oviposition cups. Within each pair, the cups were placed adjacent to one another, and pairs were spaced at 20-m intervals: 1 cup contained the treatment (fish or fish-conditioned dechlorinated tap water), and the other served as the control (dechlorinated tap water). Oviposition cups (ovicups) are 650-ml black plastic cups (14.3 cm height, 6.5 and 9 cm diameter bottom and top, respectively) filled two-thirds with dechlorinated tap water and containing a rolled germination paper as an oviposition substrate (ovistrip) that was secured to the cup’s lip with a black binder clip. Cups were punctured 5 cm below the lip to prevent overflow in case of rain. Treatments (and controls) were deployed, and ovistrips were collected 7 d later. During each weekly session, cups were checked twice and leaves or debris removed. All cups were then emptied and refilled, new fish were introduced, and ovistrips were replaced. No attractants were added to the cups. In the laboratory, eggs on the ovistrip were counted using a dissecting microscope. To confirm mosquito species identity, we flooded the ovistrips in a plastic tray and reared larvae to adulthood. All emerged adults were identified as *A. albopictus* mosquitoes.

Sub-adult *G. affinis* (3 to 4 cm in total length) were obtained from Carolina Biological Supply Company (Burlington, NC, USA).

### Experiment 1—Effect of Live Fish Presence

Dechlorinated water was added to cups. Then, a single live *G. affinis* fish was placed inside each of the treatment cups. An ovistrip was then added to the treatment and control cups. Ovistrips were collected 7 d later, and the water and the fish were replenished. The experiment was repeated 3 times (*n* = 18).

### Experiment 2—Effect of Fish-Conditioned Water

Fish-conditioned water was produced by placing 12 fish in a 1-gallon tank of dechlorinated water for 3 d following Eveland et al. (2016). Fish-conditioned water was added to the treatment cup, and 3-d-old tap water (to control for the effect of water aging) was added to the control. Water was replaced after 1 wk. The experiment was repeated 3 times (*n* = 18).

### Data Analysis

Given the paired design of this experiment (treatment and control cups coupled at each station), our data was analyzed using a 2-tailed paired *t*-test. Using the Shapiro–Wilk normality test, we confirmed that data did not significantly deviate from normality for either experiment 1 (*n* = 18, *W* = 0.961, *P* = 0.616) or 2 (*n* = 18, *W* = 0.931, *P* = 0.202). To assess the effect size, we also calculated the Oviposition Activity Index (OAI), calculated as $\mathrm{OAI}=\frac{\left(N_{t}-N_{c}\right)}{\left(N_{t}+N_{c}\right)}$. *N_t_* and *N_c_* are the numbers of eggs laid in the treatment or control cups, respectively (Kramer and Mulla 1979). OAI ranges between −1 and +1 with positive values indicating attraction to the treatment and negative values indicating aversion to it. To confirm that mean OAIs are significantly different from 0 (which would indicate no preference) we calculated the 95% confidence interval for each. In the “Fish Presence” experiment some of the cups were disturbed. We therefore repeated the above analysis for the non-disturbed and disturbed cups separately. We also tested for the effect of date (“experimental round”), and station location and their interaction with our “Test” factors (“Fish presence” or “Fish Water”) using Negative Binomial Regression generalized linear models.

## Results

### The Effect of Fish Presence

**Fig. 1.**
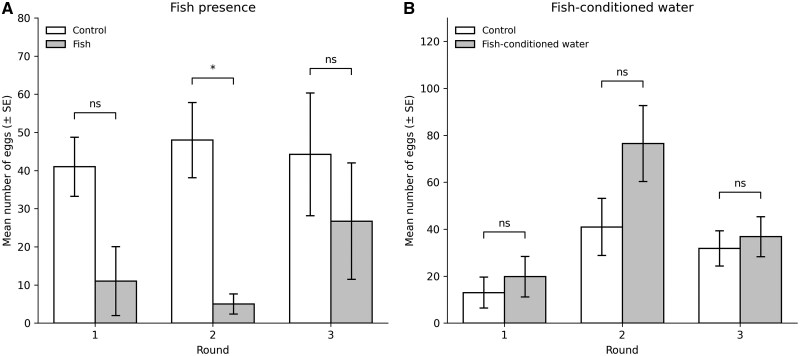
Effect of “Fish presence” versus the water control A) and “Fish-conditioned water” versus the water control B) on the mean number of eggs laid by *Aedes albopictus* during three experimental rounds. Error bars represent ± 1 SE. Asterisks (*) indicate a significant difference between treatments within an experimental round based on paired *t*-tests (*P *< 0.05); ns indicates no significant difference (*P* ≥ 0.05).

**Fig. 2.**
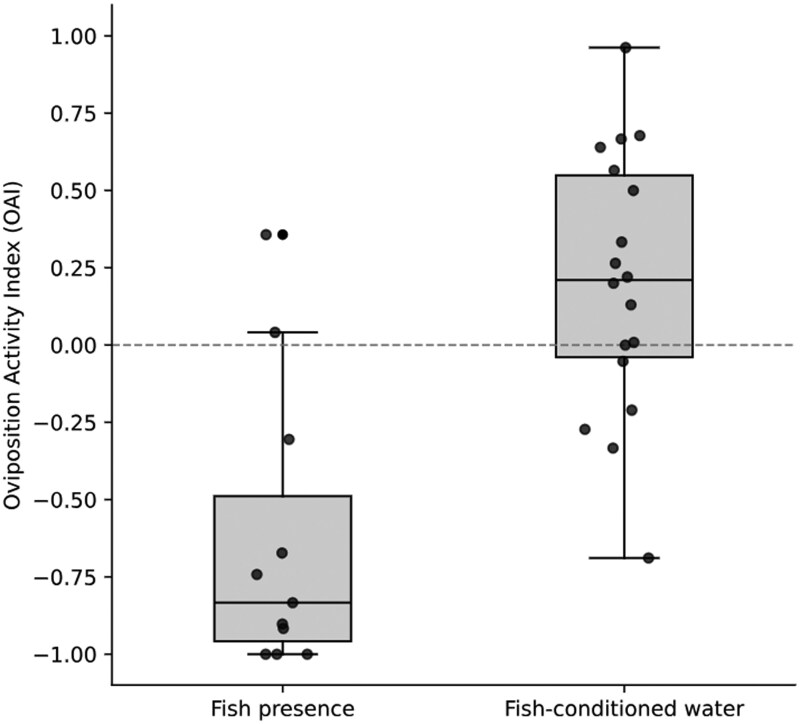
Box plots of the OAI for the effects of fish presence and fish-conditioned water on the oviposition response of *Aedes albopictus*. Positive OAI values indicate attraction to the treatment, whereas negative values indicate avoidance. The center line represents the median, the box the interquartile range (IQR), the whiskers extend to 1.5 × IQR. The dashed horizontal line at OAI = 0 indicates no preference between treatment and control. Asterisks indicate that the OAI’s 95% confidence interval (reported in the text) excludes zero, denoting a significant attraction or avoidance response.

Overall, significantly more eggs were laid in the control cups (mean ± SE: 43.2 ± 5.7, range: 0 to 92) than in cups containing fish (25.9 ± 8.2, range: 0 to 117; *t* = 2.36, df = 17, *P *= 0.03). This pattern was consistent across all 3 replicate sessions, although significant only at the second experimental round (Fig. 1A). The OAI (OAI = −0.45; 95% CI = ±0.24) of the entire data differed significantly from zero indicating a significant repellent effect of fish presence. Weekly means ranged from 29.08 (Wk 2) to 37.92 eggs (Wk 3) across experimental rounds (Fig. 1A). Overall, no significant effects of “Station” (*P* = 0.149), “Round” (*P* = 0.858) or their interactions with “Test” were observed (*P* > 0.05). Furthermore, of our 18 sampled stations, 7 were disturbed with fish missing (probably by Racoon activity). When repeating this for only the undisturbed stations, the effect of the treatment became even greater, with almost 3 times more eggs laid in the control cups compared with the treatment cups (*t* = 3.62, df = 10, *P *= 0.002) with a stronger and significant repellent effect of the treatment (OAI = −0.63, 95% CI = ±0.27; Fig. 2). In contrast, in the disturbed cups, no significant effect of the treatment was found (*t* = 0.10, df = 6, *P *= 0.92).

### The Effect of Fish-Conditioned Water

Significantly more eggs were deposited in cups containing fish-conditioned water (47.5 ± 9.1; range: 4 to 141) than in untreated water controls (30.2 ± 6.0, range: 1 to 82; *t* = 2.20, df = 17, *P *= 0.04). Although the total number of eggs varied significantly across sessions (*P* = 0.001), the trend of higher oviposition in fish-conditioned water persisted throughout the 3-wk experiment (Fig. 1B). Weekly means ranged from 16.40 (Wk 1) to 58.79 eggs (Wk 2) across the 3 experimental rounds. The OAI (OAI = 0.20; 95% CI = ±0.19) was significantly greater than zero, suggesting attraction to the fish-conditioned water (Fig. 2).

Experiment 1 (the effect of fish presence) was conducted prior to Experiment 2 (the effect of fish-conditioned water). Yet, the average number of eggs laid in the control cups did not differ significantly between the 2 experiments (*t* = 1.57, *P* = 0.126), indicating that both experiments were carried out during a comparable and suitable period of the mosquito breeding season.

## Discussion

Our results indicate a clear non-consumptive repellent effect of live fish on the oviposition response of *A. albopictus* mosquitoes. This outcome is somewhat counterintuitive, as container-breeding mosquitoes are not expected to encounter larvivorous fish in their typical oviposition sites (Vonesh and Blaustein 2010). These findings are partially consistent with those of Pamplona et al. (2009), who evaluated the effect of the physical presence of *B. splendens* and guppy (*P. reticulata*) on *A. aegypti* oviposition. In that study, Betta fish significantly deterred oviposition, whereas guppies did not, with the authors suggesting that the more aggressive foraging behavior of the Betta fish may account for this difference. Similarly, our results suggest that visual or mechanical cues from live fish (such as ripples generated by fish movement) may signal potential hazards to gravid mosquitoes. Supporting this idea, oviposition deterrence was strongest in cups that contained live fish for the full 1-wk experimental session, whereas cups from which fish were unintentionally removed (likely by racoons, based on tracks near disturbed cups) showed no significant difference in egg deposition.

Oviposition responses of mosquitoes to larvivorous fish have mostly been studied indirectly, by assessing the effects of fish-released chemical exudates on female oviposition behavior. In many cases, mosquitoes exhibited aversion to fish-conditioned water, particularly among open-water breeding genera such as *Culex* and *Culiseta* mosquitoes (Eveland et al. 2016, Segev et al. 2017, Silberbush and Resetarits Jr. 2017, Cohen and Silberbush 2021). By contrast, studies on container-breeding mosquitoes have produced conflicting results. As hypothesized by Vonesh and Blaustein (2010), container breeders are not expected to respond strongly to fish cues, as larvivorous fish rarely occur in their typical oviposition habitats. Indeed, van Dam and Walton (2008) reported that while *C. quinquefasciatus* and *C. tarsalis* avoided oviposition in fish-conditioned water, *A. aegypti* did not. However, Zuharah et al. (2016) found that both *A. albopictus* and *A. aegypti* females were repelled by kairomones released by *Hampala macrolepidota*, a predatory fish native to South-east Asia.

In our study, gravid *A. albopictus* females were not repelled; rather, they were significantly attracted to fish-conditioned water. One possible explanation is the role of microbial growth in the medium. Gravid females of many mosquito species, including *A. albopictus*, are strongly attracted to volatiles generated by microbial activity in larval rearing water (Trexler et al. 2003, Wasserberg et al. 2013, Ponnusamy et al. 2015, Girard et al. 2021). Consistent with this, *A. albopictus* has been shown to respond positively to fish-derived products (eg fish emulsion), which stimulate bacterial growth and, in turn, enhance oviposition compared with traditional hay infusions (McNamara and Healy 2022). Nevertheless, *A. albopictus* can also be repelled by bacteria associated with predators from its native range (Touray et al. 2024). We speculate that in our experiment, the attraction to fish-conditioned water may have been mediated by bacterial growth stimulated by fish excretions. Microbial abundance was not measured in this study, and further research is required to test this hypothesis.

The contrasting responses of gravid mosquitoes to direct versus indirect non-consumptive predator cues observed in our study suggest that gravid mosquitoes may balance a trade-off between resource availability (“food”) and offspring survival (“safety”) when selecting oviposition sites. The presence of a predator in an otherwise suitable aquatic habitat may generate conflicting cues: a deterrent effect from the predator itself and potentially attractive cues resulting from bacterial growth stimulated by its excretions. This trade-off may vary among mosquito species, with some species consistently prioritizing resource-rich habitats over predator avoidance (Dalal et al. 2026). In contrast, Wasserberg et al. (2013) showed that under a range of food resource levels, *A. albopictus* always avoided laying eggs in predator-(caged dragonfly nymphs) containing cups and that this perceived cost of predation was independent of resource level. Consequently, oviposition decisions reflect species-specific differences in the relative importance of larval growth opportunities versus predation risk.

Some caution should be exercised when interpreting our results regarding the direct effects of fish presence and the indirect effects of fish-conditioned water, as these 2 treatments were not done simultaneously. However, experiments were conducted 1 wk apart, and no significant difference in oviposition rate between control cups suggests that baseline conditions remained consistent across trials, reducing the likelihood of a temporal confounding effect. Additionally, although some eggs hatched during the 1-wk sessions, they remained attached to the ovistrip, and larvae were rarely observed, suggesting minimal impact on egg count estimates. Furthermore, our study does not address the underlying detection mechanism (eg chemosensory, tactile, or visual cues) or the microbial hypothesis; future studies should examine differences in microbial communities between fish and control water to identify the cues driving this behavior, and whether predator-mediated shifts in microbial composition generate trait-mediated indirect effects on aquatic community structure.

In our study, we show that the influence of aquatic predators on oviposition site selection is complex and likely multimodal, possibly involving chemosensory cues as well as visual or tactile signals. Aversion from cups containing live fish may be driven by visual or mechanical cues, such as ripples generated by fish movement, although short-term chemosensory signals from live fish may also contribute. In contrast, gravid females were attracted to water that previously contained fish (“fish-conditioned water”), likely via microbially produced cues. Together, these results suggest that *A. albopictus* mosquitoes are capable of balancing immediate predation risk against potential benefits associated with the past presence of a predator. This ability to integrate opposing cues may be widespread among other mosquito species and aquatic invertebrates that encounter variable predation and resource environments, suggesting that similar “food versus safety” trade-offs could influence oviposition and habitat selection across diverse ecological contexts.
